# Severe duodenal stenosis due to rupture of pancreaticoduodenal artery aneurysm

**DOI:** 10.1002/ccr3.1755

**Published:** 2018-08-13

**Authors:** Yoshimasa Oda, Masaki Tago, Naoko E. Katsuki, Shu‐ichi Yamashita

**Affiliations:** ^1^ Department of General Medicine Saga University Hospital Japan

**Keywords:** duodenal stenosis, pancreaticoduodenal artery aneurysm

## Abstract

When a patient complains of nausea, gastrointestinal endoscopy tends to be the first‐choice diagnostic method. However, physicians must rule out the possibility of aneurysm rupture by careful physical assessment before performing gastrointestinal endoscopy, which can be extremely dangerous.

## 1 CASE

**Figure 1:**
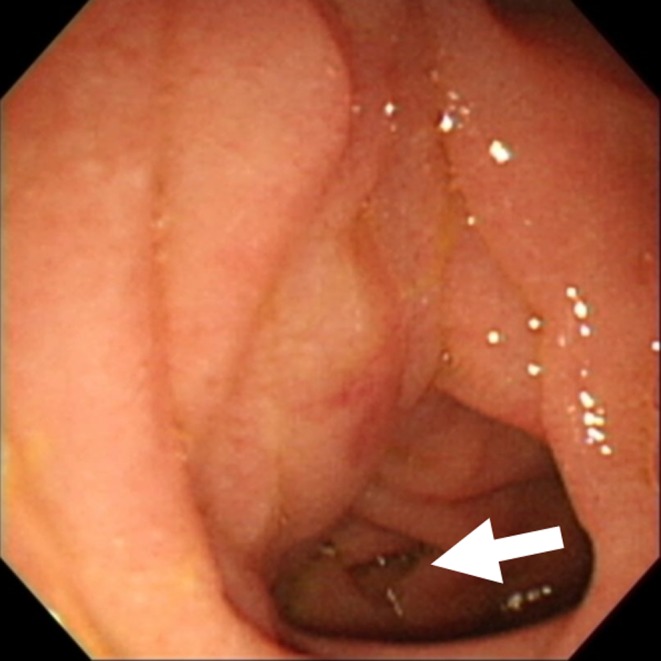
Findings of upper endoscopy. Upper endoscopy shows severe edematous stenosis at the third portion of the duodenum (arrow)

**Figure 2:**
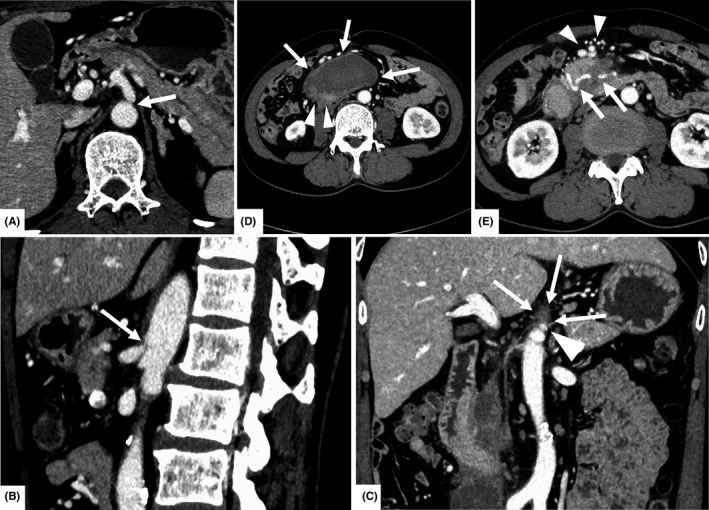
Abdominal computed tomography with contrast enhancement. A, Cross‐section image shows severe stenosis at the origin of the celiac artery (arrow). B, Sagittal section image shows severe stenosis at the origin of the celiac artery more clearly (arrow). C, Coronal section images show severe stenosis at the origin of the celiac artery (arrowhead) caused by the midline arcuate ligament (arrows). D, A 5‐ × 6‐cm low‐density mass (arrows) encloses the third portion of the duodenum (arrowheads) without definite extravasation of contrast material. E, Dilatation of the branches of the pancreaticoduodenal artery (arrows) and collateral circulation (arrowheads) is revealed.

A 57‐year‐old man with smoking history and untreated hypertension developed sudden lower abdominal pain. Although abdominal pain disappeared half a day later, abdominal bloating and nausea appeared and got worse gradually. Physical examination showed epigastric tenderness and a pulsatile, fist‐size mass under the umbilicus. Upper endoscopy showed severe edematous stenosis of the duodenum (Figure 1). Contrast‐enhanced CT revealed severe stenosis at the origin of the celiac artery caused by the midline arcuate ligament (Figure 2A‐C), a low‐density retroperitoneal mass lesion (Figure 2D), and dilatation of the branches of the pancreaticoduodenal artery (PDA; Figure 2E). Accordingly, we made the diagnosis of duodenal stenosis caused by a retroperitoneal hematoma secondary to rupture of the PDA aneurysm.

Pancreaticoduodenal artery aneurysms account for <2% of all visceral aneurysms.^1^ The mechanism of occurrence of true PDA aneurysms has been attributed to occlusion or constriction of the celiac artery caused by the median arcuate ligament, arteriosclerosis, or fibromuscular hyperplasia.^2^ We performed midline arcuate ligamentotomy to improve the stenosis at celiac artery, decreasing hematoma and clearing duodenal obstruction. The elevated pressure of the PDA might have caused formation of the aneurysm, which ruptured into the retroperitoneum, subsequently producing a hematoma.

## AUTHORSHIP

YO: involved in literature search, concept, drafting and clinical care of the patient. MT: involved in literature search, concept, and drafting. NEK: involved in drafting. SY: involved in concept and revision of article.

## CONFLICT OF INTEREST

The authors state that they have no conflict of interest.
